# Levels of *Alternaria* Toxins in Selected Food Commodities Including Green Coffee

**DOI:** 10.3390/toxins12090595

**Published:** 2020-09-15

**Authors:** Claudia Mujahid, Marie-Claude Savoy, Quentin Baslé, Pei Mun Woo, Edith Chin Yean Ee, Pascal Mottier, Thomas Bessaire

**Affiliations:** 1 Nestlé Research, Route du Jorat 57, Vers-chez-les-Blanc, 1000 Lausanne, Switzerland; claudia.mujahid@rdls.nestle.com (C.M.); marieclaude.savoy@rd.nestle.com (M.-C.S.); pascal.mottier@rdls.nestle.com (P.M.); 2 Nestlé Quality Assurance Center, 29 Quality Road, Singapore 618802, Singapore; Quentin.Basle@rdsg.nestle.com (Q.B.); PeiMun.Woo@rdsg.nestle.com (P.M.W.); EdithChinYean.Ee@rdsg.nestle.com (E.C.Y.E.)

**Keywords:** mycotoxins, *Alternaria* toxins, LC-MS/MS, isotopic dilution, compliance, tenuazonic acid, risk assessment

## Abstract

*Alternaria* toxins are emerging mycotoxins, candidates for regulation by European Authorities. Therefore, highly sensitive, confirmatory, and reliable analytical methodologies are required for their monitoring in food. In that context, an isotope dilution LC-MS/MS method was developed for the analysis of five *Alternaria* toxins (Altenuene, Alternariol, Alternariol monomethylether, Tentoxin, and Tenuazonic Acid) in a broad range of commodities including cereals and cereal-based products, tomato-based products, tree nuts, vegetable oils, dried fruits, cocoa, green coffee, spices, herbs, and tea. Validation data collected in two different laboratories demonstrated the robustness of the method. Underestimation of Tenuazonic Acid level in dry samples such as cereals was reported when inappropriate extraction solvent mixtures were used as currently done in several published methodologies. An investigation survey performed on 216 food items evidenced large variations of *Alternaria* toxins levels, in line with data reported in the last EFSA safety assessment. The analysis of 78 green coffee samples collected from 21 producing countries demonstrated that coffee is a negligible source of exposure to *Alternaria* toxins. Its wide scope of application, adequate sample throughput, and high sensitivity make this method fit for purpose for the regular monitoring of *Alternaria* toxins in foods.

## 1. Introduction

The ability of fungi to produce mycotoxins is largely influenced by temperature, rainfalls, relative humidity, and stress conditions in the plants. Mycotoxins are therefore one of the most severe food safety hazards amplified by global climate change, which may facilitate appearance and dissemination of new toxins, the so-called “emerging mycotoxins” [1]. Among them, *Alternaria* toxins are secondary metabolites produced by *Alternaria* fungal species, most commonly *Alternaria alternata* but also *Alternaria tenuissima* and *Alternaria infectoria*. As pathogens, they affect many crops including grains, oil seeds, spices, and various fruits and vegetables, and may thus enter the food chain. Due to their growth even at low temperature, *Alternaria* fungal species are also responsible for spoilage of commodities during refrigerated transport and storage [2,3,4].

*Alternaria* fungal species produce more than 70 toxins but only a small number of them have been chemically characterized so far [5]. They exhibit broad structure divergence and are commonly divided into five different classes. Some toxins such as alternariol (AOH), alternariol monomethyl ether (AME), tenuazonic acid (TeA), and altertoxins were described to induce harmful effects in animals, including fetotoxic and teratogenic effects. Culture extracts of *A. alternata* but also AOH and AME were found mutagenic and clastogenic in various in vitro systems [2,3]. Considering their possible harmful effects, presence of these toxins in food may be considered as a serious threat to public health.

These findings prompted the European Food Safety Agency (EFSA) to publish a first risk assessment on *Alternaria* toxins (TeA, AME, AOH, Tentoxin (TEN), and Altenuene (ALT)) in feed and food in 2011 [3]. Due to the lack of toxicity data available, the expert panel could not establish health-based guidance values and used the threshold of toxicity concern (TTC) for risk assessment. The TTC concept holds that the presence of a chemical in food does not represent a safety concern even in absence of toxicological data, provided exposure remains below the TTC of the pertinent structural class [3,6]. Based on the limited analytical data available at that time, the estimated mean chronic dietary exposures exceeded TTC values for the two genotoxic compounds AOH and AME. A dietary exposure assessment to *Alternaria* toxins in the European population was then published 5 years later [4], using thousands of data generated during the 2010–2015 period [7]. Interestingly, higher exposures were estimated compared to those estimated in 2011, and highest levels were reported for TeA with tomato-based products, tree nuts, oil seeds, grains, and fruits being the most contaminated commodities. It was also noted that infants were the population group with the highest dietary exposure. EFSA still recommended to develop sensitive analytical methods to allow quantification at low levels (avoiding too many left-censored data) and to generate more analytical data in foodstuffs.

In this context, various methodologies and studies on *Alternaria* toxins in foods and drinks have been published over the last years, e.g., in tomato-based products, fruit and vegetable-based products, wine [5,8], grapes [9], tea and herbal infusions [10], dried figs, olives, sunflower seeds and cereals [11], wolfberry [12], drinking water [13]. A study from the Technical University of Munich [14] has showed that infant cereals and baby food purees purchased from German supermarkets were frequently contaminated with *Alternaria* toxins. AME, TEN, and TeA were found in more than 80% of the samples at levels up to 1.4 (AME), 7.5 (TEN), and 221 µg/kg (TeA). In the latest study by Gambacorta, et al. [15], a large proportion of the 94 spices and 37 herbs analyzed were found contaminated with several *Alternaria* toxins. TeA was the predominant mycotoxin with the highest percentages of positive samples (76%), followed by AME (46%), TEN (37%), AOH (34%), and ALT (5%) with maximum levels up to 106,793, 306, 179, 636, and 22 μg/kg, respectively. The EFSA database and recent scientific publications did not identify coffee beans as a significant target of *Alternaria* contamination. Occurrence of these toxins in green coffee was investigated in one single study in which low levels of AOH and AME were reported [16]. However, ALT and TeA were not considered for analysis.

No regulation applies for *Alternaria* toxins in foodstuffs yet, but maximum levels are currently under consideration by the European Commission [17]. In June 2019, a draft EU Commission Recommendation on the monitoring of three *Alternaria* toxins (AOH, AME, TeA) in food was issued which included benchmark values above which investigations by Food Business Operators would be appropriate to identify the factors resulting in high levels in certain foods [18]. Benchmark values in processed tomato products, sesame seeds, sunflower seeds, sunflower oil, but also in cereal-based foods for infants and young children, were set at levels ranging from 5 to 30 µg/kg (AOH, AME) and 100–500 µg/kg (TeA). Benchmark levels were set as well for TeA in tree nuts (100 µg/kg), dried fruits (1000 µg/kg), and paprika powder (10,000 µg/kg). As stated in the draft document, these benchmark values do not represent safe levels in food.

Therefore, there is a need for reliable analytical methodologies applicable to a broad range of food commodities. In that context, the European Commission Joint Research Centre (JRC, Geel, Belgium), which was the EU Reference Laboratory for Mycotoxins at that time, organized a Multi Laboratory Test (MLT) in 2018 to support a LC-MS/MS method standardization by the European Committee for Standardization [19,20]. Although limited to the analysis of tomato purees, wheat flour, and sunflower seeds, its analyte scope (AOH, AME, TeA, TEN, and ALT) and quantification approach (isotope dilution) made this confirmatory method a good candidate for deployment in high routine environments, facing today a broad range of items to monitor, an increasing pressure to shorten turn-around time, and cost constraints [21]. Already published methodologies did not match with these expectations due to (a) limited number of food items considered [20], (b) limited analyte scope missing important *Alternaria* toxin(s) [11], (c) cumbersome sample extraction [14], or (d) quantification approach hardly workable for “multi-matrix” analytical method [8,15,22,23,24].

Consequently, the method validated during the MLT was tentatively extended to a broader range of food commodities including all the ‘EU benchmarked foods’. The present study describes the modifications that were brought to circumvent the analytical issues observed. Extensive validation data collected in two different laboratories are presented to demonstrate method robustness. This study also aims to provide additional data to get a reliable picture of the occurrence of *Alternaria* toxins in common foods including green coffee for which analytical data are scarce. A risk assessment was further performed to evaluate whether coffee could be a significant source of exposure to *Alternaria* toxins.

## 2. Results and Discussion

### 2.1. Analytical Method Development

#### 2.1.1. LC-MS/MS Conditions

Detection of the five *Alternaria* toxins were first evaluated using either positive or negative electrospray ionization modes. AOH, AME, and TeA exhibited a predominant deprotonated molecular ion [M − H]^−^ whereas TEN and ALT were seen either as [M + H]^+^ or [M − H]^−^ species. To avoid the use of polarity switching that may hamper method performances when using older LC-MS instruments, the negative ionization mode was exclusively selected as done previously [20]. Collision-induced dissociation (CID) mass spectra of each precursor were then recorded at various collision energies before selecting the optimal fragment ions, MS/MS transition reactions, and related instrument parameters. MRM transition reactions of each internal standard (IS) were the ones corresponding to the related native toxin taking into account the degree of isotope labeling. Adequate LC separation of the five *Alternaria* toxins was attained with a classical Waters Acquity BEH C18 column employing a 11-min LC gradient in alkaline conditions as done in other studies [5,8,20,21,23]. Mobile phases were ammonium acetate (5 mM) in water pH 8 and methanol. TeA (the most polar compound) eluted at retention time 2.0 min, comfortably longer than the void volume of the column, followed by ALT, AOH, TEN, and AME at 3.5, 3.8, 4.3, and 5.0 min, respectively.

#### 2.1.2. Sample Preparation

*Alternaria* toxins were initially extracted following an analytical procedure (namely MLT method) evaluated in 2018 during a JRC intercollaborative study. Briefly, wheat flour, tomato puree, and sunflower seeds were extracted with a methanol/water/acetic acid (85/14/1, *v/v/v*) mixture, and the extracts subsequently purified on a polymeric solid-phase extraction (SPE). To point out that a large proportion of organic solvent is used in such extraction medium (i.e., 85% in that case), a fact recurrently noticed in other published methodologies, e.g., methanol/water (80/20, *v*/*v*, [20]), acetonitrile/water/formic acid (80/20/0.1, *v/v/v*, [8] or 84/16/1, *v/v/v*, [11]), methanol/water/acetic acid (79/20/1, *v/v/v*, [23]).

Applicability of the QuEChERS (Quick, Easy, Cheap, Efficient, Rugged and Safe) approach for the analysis of *Alternaria* toxins was considered in parallel since already validated elsewhere [8,9,25]. This approach is faster, user friendly, cost efficient, and already used in many laboratories dealing with chemical contaminants analysis, e.g., mycotoxins, veterinary drugs, pesticides [26,27,28,29]. Our protocol encompasses an initial extraction of the analytes with a mixture of water, acetonitrile, and formic acid (50/50/0.1, *v/v/v*), followed by a liquid–liquid partition using salt mixtures.

Both MLT and QuEChERS approaches were compared using fortified tomato purees, sunflower seeds, and cereal samples but also incurred commodities (cereals). Similar results were obtained for ALT, AOH, AME, and TEN. Surprisingly, underestimation of TeA level was observed using the MLT method applied for dry incurred matrices (e.g., cereals), but not for high-water content samples (e.g., tomato puree) or in fortified samples, these latter generally used to mimic natural contamination during method validation and/or quality control plans. Recovery of the related labeled internal standard (IS) ^13^C_2_-TeA spiked on the test portion at the beginning of the procedure was around 80% by both methods demonstrating that no matrix effects occurred and that the overall extraction of the IS worked well. In our hands, these data suggested that discrepancies between both approaches originated from the extractability of the native TeA toxin.

**Figure 1:**
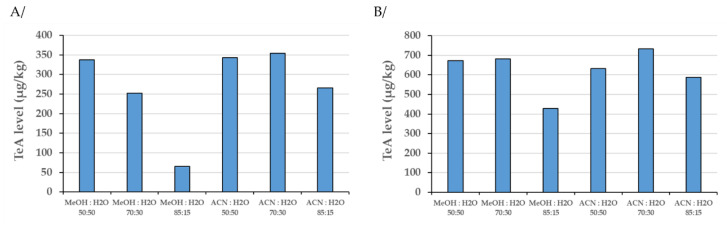
Impact of the water content in extraction mixtures on tenuazonic acid (TeA) levels in incurred samples ((**A**) breakfast cereal; (**B**) rice flour). All extraction mixtures were supplemented with 0.1% formic acid.

The impact of water content in the extraction mixture was thus investigated using a cereal-based product (mix of seven cereals: rice, millet, spelt, oat, rye, barley, and corn) and a rice flour, both known to contain ‘natural’ levels of TeA. Samples were extracted with mixtures containing either acidified methanol or acetonitrile, each with different amounts of water. Extracts were first thoroughly shaken, diluted, then centrifuged, and subsequently injected into the LC-MS/MS. As shown in Figure 1, an incomplete extraction of the native TeA toxin was observed when extraction mixtures contained only 15% of water, as considered in the MLT method. These data strongly suggest that TeA, the most frequently detected *Alternaria* toxin, needs a certain amount of water to be efficiently extracted from incurred dry samples.

Historically, most methods for monitoring *Alternaria* toxins were devoted to the analysis of high-water content commodities such as tomatoes and tomato-based products and fruit juices [24,25,30,31]. In that case, the low amount of water in the extraction mixture might be compensated by the natural water content of the matrix. However, an efficient method for such monitoring should be applicable to a broad range of foods whatever their water content, this with the goal to fulfill EFSA recommendations for data collection. In that context, our initial findings prompted us to favor the development of a “multi-matrix” QuEChERS-based method rather than an extension of the MLT method to other matrices.

Optimization of the QuEChERS approach was conducted using samples known to be challenging when analyzed by LC-MS/MS, e.g., green coffee and spices, due to strong signal suppression (i.e., matrix effects). Best results were obtained using Na_2_SO_4_/NaCl (4 g + 1 g) salts to induce liquid–liquid partition and hexane to remove co-extracted fat as a subsequent clean-up step. This clean-up procedure is extensively used for mycotoxins and tropane alkaloids analyses in Nestlé laboratories [26,28,32]. To reduce the strong matrix effects observed for ALT and AOH in some matrices, smaller test portions were considered for tree nuts, dried fruits, green coffee, cocoa, tomato-based products, and vegetable oils (2.5 g) and for spices, herbs, and tea (0.5 g).

Quantification was finally conducted by isotopic dilution using labeled isotopomers for each of the five *Alternaria* toxins as internal standards.

### 2.2. Method Performance Characteristics

The method was first validated by Laboratory 1 (Switzerland) for cereals and cereal-based products, tomato-based products, and spices at two fortification levels and for tree nuts, vegetable oils, and dried fruits at a single fortification level using a sample representative of each commodity group. The five solvent-based calibration curves followed a linear model with r^2^ > 0.99 over the 0–100 ng/mL range (0–500 ng/mL for TeA). Deviations of back-calculated concentrations of calibrants standards from the true concentration were never out ±20%, as requested by SANTE/12682/2019 document [33].

As shown in Table 1 and Table 2, analytical performance data were RSD(r) < 12%, RSD(iR) < 15% and recoveries ranging from 89% to 112% regardless of the toxin/matrix/fortification levels, thus fulfilling SANTE/12682/2019 requirements [33]. Only TeA precision data in spices were slightly higher due to the unavailability of TeA-free paprika powder samples. Although the lowest TeA content sample was selected for validation purpose, performance data generated at the 1250 µg/kg spiking level (RSD(r): 26% and RSD(iR): 29%) were still impacted by the native TeA content (4500 µg/kg) of this item. This precluded the collection of validation data at low level (i.e., 50 µg/kg) in this commodity in Laboratory 1. Interestingly, this incurred paprika powder was also analyzed as such (i.e., without fortification) in duplicate over 6 days leading to RSD(r) and RSD(iR) values at 3.7% and 2.9%, respectively, evidencing the low test portion size (0.5 g) as sufficiently representative when dealing with such powdered sample. Applicability of the method to other food commodities was further demonstrated by analyzing additional samples in duplicate, each after fortification, e.g., at 0.5 µg/kg for green coffee (2.5 µg/kg for TeA), at 2 µg/kg for cocoa powder (10 µg/kg for TeA), and 10 µg/kg for tea and herbs (50 µg/kg for TeA). Differences between duplicates were <20% and recovery data ranged from 75% to 119%.

Performance parameters including sensitivity, precision, and recovery were then further confirmed by Laboratory 2 (Singapore), ultimately demonstrating ruggedness of the method.

Limits of quantification (LOQs) were arbitrarily set for each matrix/toxin combination at the lowest validated levels and ranged from 0.5 to 10 μg/kg for ALT, AOH, AME, and TEN and from 2.5 to 50 μg/kg for TeA (Table 1).

Over time, stability of toxin standards in solutions is key information which is systematically challenged during accreditation and GLP assessment audits. Both labeled and unlabeled stock standards solutions stored in the freezer at −20 °C were thus compared one year later against freshly prepared ones within the same analytical sequences i.e., under repeatability conditions to circumvent inherent day-to-day variations of mass spectrometry measurements. All stock solutions (100 µg/mL in methanol) were found stable for at least one year. As well, sample extracts were found stable for up to 4 days after extraction when stored in the fridge at 4 °C or in the autosampler cooled down at max 8 °C. Such information is valuable, typically when the extracted solutions need to be reinjected in case of instrument shutdown, aborted sequence, etc.

### 2.3. Occurrence of Alternaria Toxins in Various Food Commodities

A summary of *Alternaria* toxins levels found in each sample category (corresponding to 138 samples in total) is given in Table 3 Individual data along with sample details are available in Table S1.

Spices (*n* = 21) analyzed were caraway, coriander, garlic, chili, cayenne pepper, and paprika. EU benchmark level has been set only for TeA in paprika powder at 10,000 μg/kg. In our survey, only one paprika powder (TeA 7356 μg/kg) over the eight analyzed was found below this benchmark level (concentrations in outliers: 10,163–18,856 μg/kg). Chili items were found quite contaminated by TeA as well, with concentrations ranging from 4510–20,478 μg/kg. Co-occurrence of several *Alternaria* toxins (but not ALT) was frequently observed in this ingredient category, and at the highest concentrations found in this survey (exception was garlic in which none of the toxins were detected).

Herbs (*n* = 11) were marjoram, oregano, and thyme. Again, TeA was found in all items (but one) at levels up to 748 μg/kg, and ALT was not detected at all. Other toxins ranged within the <10–113 μg/kg concentration range. Such high concentrations of *Alternaria* toxins in spices and herbs are aligned with data reported in a recent publication [15].

Levels of *Alternaria* toxins in sunflower oils (*n* = 4) were all below the EU benchmark levels set for this material, which are 10 μg/kg for AOH, 10 μg/kg for AME, and 100 μg/kg for TeA. TEN (not a benchmarked toxin) was detected in one sample at 3.9 μg/kg, and AME in another one at 2.4 µg/kg. Recently, 16 different sunflower oil samples were analyzed, and only TeA and TEN were detected (in one and three samples, respectively) at levels <10 µg/kg [20]. In another study [23], TeA was detected in 31% of 39 sunflower oils at level up to 458 µg/kg (mean of positive values 163 µg/kg). The authors reported that cold-pressed sunflower seed oils showed significantly higher *Alternaria* toxins contaminations compared to refined products. Interestingly, all these studies highlighted TEN to be the most prevalent toxin in sunflower seed oils, albeit quantified at low levels (<10 µg/kg).

Tree nuts (*n* = 13) comprised almonds, hazelnuts, peanuts, and pistachio. A benchmark level at 100 μg/kg for TeA has been set for this food category. None of the samples under survey reached this value (highest level was 62 μg/kg). Other toxins (with the exception ALT) were sporadically detected (<2–6.4 μg/kg).

Vegetables (*n* = 14) were carrot and pea (processed ingredients), and tomato-based products (powder, pulped, sauce, etc.). Only ‘processed tomato products’ is mentioned in the EU draft document with limits set at 10 μg/kg for AOH, 5 μg/kg for AME, and 500 μg/kg for TeA. Two tomato products were found ‘not compliant’ in regard to these benchmark levels: one containing AOH (13.7 μg/kg) and TeA (641 μg/kg), and the second containing AOH (65.3 μg/kg), AME (7.9 μg/kg), and TeA (1096 μg/kg). Occurrences of high levels of TeA in fresh and dried tomatoes in Italy have been recently reported [34].

Cereals (*n* = 31) surveyed were corn, barley, millet, oat, rice, rye, rapeseed meal, sorghum, soya, spelt, and wheat. TeA was evidenced in most items at levels ranging from 3.5 to 766 µg/kg. Highest levels were observed in a rice flour (758 µg/kg) and two rapeseed meal samples (766 and 636 µg/kg). Other *Alternaria* toxins, with the exception of ALT, were sporadically detected at levels <31 μg/kg.

Cereal-based products (*n* = 15) comprised biscuits or breakfast cereals that were collected in local supermarkets. As highlighted for cereal samples, TeA was evidenced in most items at levels ranging from 5.2 to 628 µg/kg. One biscuit and two breakfast cereals were found with both AOH and AME at levels <10 µg/kg. TEN, not a benchmarked toxin, was often found in these foods but at low levels (max. 6.9 µg/kg).

Food commodities not mentioned in the EU draft recommendation were also considered to get insight about occurrence of *Alternaria* toxins. Fruits (*n* = 9) comprised dried fruits (apricots, raisins, and blueberries) and a single pomegranate juice. All dried fruits contained TeA at levels from 33 to 158 μg/kg with the exception of dried raisins. Other toxins were at <20 μg/kg. Wei et al. [35] already alerted about the high incidence rate of TeA (but also of AOH, AME, and TEN) in dried fruits from China (wolfberries, apricots, raisins, and figs). The pomegranate juice concentrate was contaminated by all five toxins at levels largely above those depicted in the other fruit-based items, i.e., at 17, 80, 48, 13, and 685 μg/kg for ALT, AOH, AME, TEN, and TeA, respectively.

To our knowledge, the presence of *Alternaria* toxins in cocoa (cocoa nibs, cocoa powder) and dried tea (black tea, green tea, etc.) has not been investigated previously. None of the toxins were detected in the *n* = 5 cocoa items and *n* = 15 tea samples surveyed at our LOQs.

From a global perspective, the data collected on these 138 samples are well aligned with the last EFSA reports. ALT was detected in one out of the 138 samples surveyed (0.7%), confirming previous EFSA findings where only 0.6% of samples were reported positive (mainly oilseeds) [3]. It is important to mention that ALT is not cited anymore in the 2016 EFSA report [4]. On the contrary, TeA was the most often detected *Alternaria* toxin in this set of raw materials and finished products (62%), and at the highest concentrations whatever the food material. Typically, paprika and chili powder were prone to contamination with levels regularly depicted above 10,000 µg/kg. AOH, TEN, and AME toxins were detected in 33%, 30%, and 21% of the samples with large discrepancies between commodities, for which spices, herbs, fruits juices, and cereals represented the majority of positive findings. In total, nine samples were above the still discussed EU benchmark levels, due to exceeded levels of TeA, AOH, and/or AME in paprika samples and tomato products. It is worth mentioning that one or several *Alternaria* toxins were concomitantly depicted in several commodities tested, with large variability in concentrations. However, no co-occurrence trend can be extrapolated from our limited set of data.

### 2.4. Occurrence of Alternaria Toxins in Green Coffee

A summary of *Alternaria* toxins levels in green coffee, sorted by country of origin, is given in Table 4 (full details available in Table S1). In the large majority of samples, none of the five targeted *Alternaria* toxins were detected. Only seven samples (9%) were found positive with at least one toxin, and were from Brazil (*n* = 1), Peru (*n* = 1), Ivory Coast (*n* = 1), and Vietnam (*n* = 4). AOH was detected in four samples at levels <3 μg/kg. AME and TEN were each quantified in one single sample at level <1.3 μg/kg and TeA in two samples (10 and 13 μg/kg). ALT was not detected in any sample. Co-contamination at quantifiable levels was recorded in one single sample from Vietnam (AOH + AME, 2.98 µg/kg).

Both occurrence and levels of individual *Alternaria* toxins in green coffee were therefore low in this study. This holds even for TeA which was the most frequently found toxin and at highest levels in the other commodities considered. Due to the very low occurrence of *Alternaria* toxins, a potential relation between the country of origin and contamination levels could not be investigated further.

In a previous study conducted on 85 green coffee samples from nine countries [16], AME was more frequently detected and quantified (seven samples, 8%) at levels ranging from 0.6 to 8.3 μg/kg. AOH and TEN were not seen but LOQs were much higher at that time (10 and 5 µg/kg, respectively). TeA and ALT were not analyzed.

### 2.5. Risk Assessment: Alternaria Toxins in Green Coffee

As mentioned earlier, the five EFSA selected *Alternaria* toxins are not well characterized from a toxicological point of view [3]. ALT, TeA, and TEN were not found mutagenic in the in vitro bacterial Ames test [3,36]. No data on genotoxicity in mammalian systems are available. Thus, all of them belong to the structural Cramer class III with a TTC of 1500 ng/kg BW/d [3,6]. On the contrary, AME and AOH were found mutagenic in the bacterial Ames test. Only AOH was found genotoxic in mammalian cells in vitro. AOH was not genotoxic in the in vivo micronucleus test (bone marrow) and in the in vivo alkaline comet assay (liver), probably because systemic AOH bioavailability is very low. Local genotoxicity at the site of tissue contact (gastrointestinal tract) could not be adequately analyzed because of adverse effects of repeated corn oil gavage [37]. Based on the available data, a genotoxic/carcinogenic potential of AOH on the mucosa of the gastrointestinal tract can be assumed. EFSA concluded that it was appropriate to apply the TTC of 2.5 ng/kg BW/d for genotoxic carcinogens. Similarly, TTC for genotoxic carcinogens was applied to AME.

An adult of 60 kg body weight drinking five cups of coffee per day was taken as reference exposure scenario covering the majority of consumers. The manufacturing of coffee from green coffee comprises roasting loss, extraction into the brew, as well as processing factors for soluble coffee. Therefore, a consumption equivalent of 28 g of green coffee per day was considered, covering all types of coffee products.

The principal source of mycotoxins in food are usually cereals, a major staple food. The TTC refers to total intake from all potential sources. In order to avoid total dietary exposures exceeding the TTC, *Alternaria* toxins in coffee should contribute only to a small proportion of the total exposure. Up to 10% of the TTC as contribution by mycotoxin exposure from coffee was considered to be without safety concern. Considering the scenario exposure (28 g/d green coffee), the 10% threshold level corresponds to safe levels of 0.54 µg/kg of AOH/AME and 320 µg/kg of TeA/TEN/ALT in green coffee. The LOQs achieved in this method for coffee (0.5 µg/kg, except TEA at 2.5 µg/kg) are thus low enough to ensure that not quantifiable levels can a priori be considered as safe and to focus the risk assessment on positive findings.

Exposures were calculated for the highest level of each toxin analyzed which represents a worst-case scenario. AOH and AME being structurally similar, and both mutagenic and genotoxic, their toxicity is expected to be additive in case of combined exposure. In the single case of co-contamination, exposure from the sum of AOH and AME was compared to the TTC for genotoxic carcinogen.

As shown in Table 5, exposure to the carcinogenic AOH, AME, and AOH + AME from coffee could represent much more than 10% of the relevant TTC whilst exposure to the non-genotoxic molecules ALT, TEN, and TeA was negligible. However, the TTC value is not a threshold to adverse effects but rather a guideline for the level of concern and based on very conservative assumptions. The true carcinogenic potency of *Alternaria* toxins in mammals is not known. Based on EFSA evaluation, the estimated chronic exposure to these toxins from the diet (which did not include coffee) is about 3× the TTC even on average and may be >10× the TTC in high consumers [1,3]. Additional exposure from coffee, even with co-contamination, would not significantly increase total exposure. Perhaps most importantly, this risk assessment was performed on green coffee without considering the impact of roasting. The fate of *Alternaria* toxins upon coffee roasting is unknown. Published information is available essentially for Ochratoxin A, Aflatoxins, and Sterigmatocystin, for which destruction by roasting of about 80%, 50–60%, and 70%, respectively, were reported [38,39,40,41]. Such information suggests that substantial destruction of several structurally different mycotoxins occurs during roasting. Therefore, even the highest calculated exposures to *Alternaria* toxins from green coffee beans are not considered to represent a safety concern for consumers: they are sporadic and short-term whereas TTC refers to regular chronic exposure over a lifetime.

## 3. Conclusions

The underestimation of Tenuazonic acid in dry samples such as cereals and cereal-based products was reported when inappropriate extraction solvent was used as currently done in several methodologies. Such findings prompted us to develop our own isotope dilution confirmatory LC-MS/MS method for the analysis of five *Alternaria* toxins in raw materials comprising cereals, cocoa, green coffee, fruits, herbs, nuts, sunflower oils, spices, tea, and vegetables, but also in some finished products. Validation data collected in two different laboratories unambiguously demonstrated the robustness of the method.

Occurrence data on 216 samples has evidenced the presence of one or several toxins in almost all food commodities tested. Cocoa, tea, and sunflower oil seem to be less affected by *Alternaria* fungal species, but more data would be needed to confirm this statement. Tenuazonic acid was the most often detected *Alternaria* toxin, and at the highest concentrations whatever the food material. Typically, paprika and chili powder were prone to contamination with levels regularly depicted above the 10,000 µg/kg EU benchmark level for paprika. Due to the lack of data available in coffee, the analysis of 78 samples of green coffee from all producing regions worldwide was included in this study. Contamination of green coffee beans by *Alternaria* toxins was rare and low. It is important to note that the fate of *Alternaria* toxins upon coffee roasting is still unknown. Our data suggest that coffee is a negligible source of exposure to *Alternaria* toxins and that there is no safety concern.

The wide scope of application of this analytical approach along with its adequate sample throughput at µg/kg level make it fit for purpose for regular monitoring of *Alternaria* toxins in foods in high routine environments.

## 4. Materials and Methods

### 4.1. Chemicals and Reagents

LC gradient grade solvents (acetonitrile, methanol, n-hexane, isopropanol) and water were from Merck (Darmstadt, Germany) whereas concentrated formic acid, 25% ammonia solution, and ammonium acetate were purchased from Sigma-Aldrich (Buchs, Switzerland). Ready-to-use QuEChERS salts containing 4 g of sodium sulphate (Na_2_SO_4_) and 1 g of sodium chloride (NaCl) were from Agilent (Geneva, Switzerland).

*Alternaria* toxins, namely altenuene (ALT), alternariol (AOH), alternariol monomethyl ether (AME), tentoxin (TEN), and tenuazonic acid (TeA) were obtained from ASCA GmbH (Berlin, Germany). Related isotopically labeled standards, which are ^2^H_6_-Altenuene (ALT-IS), ^2^H_3_-Alternariol (AOH-IS), ^2^H_3_-Alternariol monomethylether (AME-IS), ^2^H_3_-Tentoxin (TEN-IS), and ^13^C_2_-tenuazonic acid (TeA-IS) were from ASCA GmbH as well, each with chemical purity >99% and isotopic purity >97%.

### 4.2. Preparation of Standard Solutions

Stock standard solutions of each unlabeled and labeled toxin were prepared at 100 µg/mL in methanol. Composite working solutions of unlabeled standards were then obtained by successive dilutions at 1 (TeA at 5 µg/mL) and 0.1 µg/mL (TeA 0.5 µg/mL) using water methanol (80 + 20) as solvent mixture. A composite working solution of the internal standards (ISs) was prepared at 1 µg/mL (5 µg/mL for TeA-IS) in methanol. All solutions were stored at −20 °C and attained room temperature (RT) before use.

### 4.3. Sample Collection

Food raw materials (*n* = 116) were collected from own facilities and comprised nuts (13), dried fruits or fruit juice concentrate (9), sunflower oils (4), herbs (11), spices (21), cocoa (5), vegetables (11), tea (15), and cereals (27). Commercially available foodstuffs (*n* = 22) were purchased from local supermarkets in Switzerland and were cereal-based products (breakfast cereals (8), biscuits (7)), rice (4), and tomato products (3; puree, ketchup, and dried).

In addition, green coffee samples (*n* = 78) were obtained from suppliers located in 21 producing countries which are Vietnam, Ivory Coast, Brazil, Colombia, Ethiopia, Honduras, Indonesia, Papua New Guinea, Peru, Uganda, Mexico, Cameroon, China, Costa Rica, Guatemala, India, Kenya, Nicaragua, Philippines, Rwanda, and Thailand.

When the sample was not in powdered form with homogeneity visually not sufficient, intensive comminution (at least 100 g) was performed using a cryogenic grinder (SPEX 6875D Freezer/Mill, Stanmore, UK) and the ground sample kept in an air-tight container at −20 °C until analysis.

### 4.4. Sample Extraction Methodology

The finely ground test portion or oil (5 g for cereals and cereal-based products and green coffee, 2.5 g for tree nuts, dried fruits, cocoa, vegetables included tomato-based products, and vegetable oils, or 0.5 g for spices, herbs, and tea) was weighed into a 50-mL polypropylene tube (Becton Dickinson, Le Pont de Claix, France) to which 50 µL of the composite IS working solution at 1 µg/mL (5 µg/mL for TeA-IS) was added. The IS fortified sample was thoroughly mixed on a vortex to ensure that the spiked volume was totally absorbed by the matrix. Water (10 mL) and a ceramic homogenizer (Agilent, Geneva, Switzerland) were added and the tube vigorously shaken until no lumps were present. Acetonitrile containing 0.1% concentrated formic acid (10 mL) was then added and the mixture shaken using a mechanical shaker (SPEX SamplePrep GenoGrinder, Stanmore, UK) at 1500 rpm for 3 min. QuEChERS salts (5 g) were supplemented to the slurry which was shaken again using the GenoGrinder shaker (1500 rpm, 3 min). After centrifugation at 4000× *g* for 10 min at room temperature (Heraeus Multifuge, Thermo Fisher Scientific, Bremen, Germany), the supernatant (5 mL) was transferred into a 15-mL polypropylene tube to which hexane (5 mL) was added for defatting purpose. The mixture was first mechanically shaken (GenoGrinder 1500 rpm, 3 min), then centrifuged (4000× *g*, 1 min). The upper hexane phase was discarded and the lower phase (1 mL) transferred into a new 15-mL polypropylene tube. After evaporation to dryness under a stream of nitrogen at 40 °C, the residue was resuspended in water:methanol (80 + 20, 0.5 mL) by vortexing (5 s) and sonication (3 min), and eventually centrifuged at 13,000× *g* at room temperature for 10 min using a benchtop centrifuge (Heraeus Frisco 17, Thermo Scientific). If still cloudy, the supernatant was filtered by means of a hydrophilic PTFE 0.20 µm syringe filter (Millex-LG, Sigma-Aldrich). The final extract was collected into a HPLC amber glass vial for further LC-MS/MS analysis.

Initial trials were performed using the method developed for the MLT. In that case, the finely ground test portion (2 g) was extracted with 15 mL of methanol/water/acetic acid (85/14/1, *v/v/v*) mixture. After shaking (45 min) and centrifugation (10 min, 4000× *g*), the supernatant (7.5 mL, equivalent to 1.0 g sample) was diluted with an equal volume of 1% (*v/v*) aqueous acetic acid solution before being purified on a polymeric solid-phase extraction (SPE) cartridge (Phenomenex Strata-XL, 6 cc, 200 mg). After evaporation (7 mL), the extract was first reconstituted with 400 μL of methanol, extensively vortexed for 20 s, and completed with 600 µL of a 5 mM ammonium acetate buffer (pH 8.0). After filtration using a PTFE syringe filter (Millex-LG), the sample was injected onto a LC-MS/MS instrument.

### 4.5. LC-MS/MS Methodology

Liquid chromatography was performed on an Agilent 1290 Infinity system (Geneva, Switzerland) using a VanGuard BEH C18 pre-column (2.1 × 5 mm, 1.7 µm) attached to a Acquity UPLC BEH C18 column (2.1 × 100 mm, 1.7 µm), both from Waters Corporation (Milford, MA, USA) and thermostated at 50 °C. Mobile phases were composed of ammonium acetate 5 mM in water pH 8 (solvent A) and methanol (solvent B). A gradient program was set up as follows: 0–0.2 min with 95% A; 0.2–2 min linear gradient down to 50% A; 2–6 min linear gradient down to 0% A, hold at 0% A for 2.5 min; return to 95% A in 0.1 min and hold at 95% A for 2.4 min (total run time 11 min). Injection volume was 5 μL and the autosampler temperature was set at 8 °C. The 0.4 mL/min flow was directed into the MS detector between 1.5 and 6.5 min using a diverter valve. MS detection was performed using Sciex TRIPLE QUAD 6500+ instruments (Foster City, CA, USA) equipped with a Turbo V™ Ion Source. MS parameters were obtained in negative electrospray ionization (ESI) mode by separately syringe-infusing each individual standard solution at 0.1 µg/mL (syringe flow rate of 10 µL/min) along with the LC flow at 0.4 mL/min using a T connector. The LC flow was constituted of 50% aqueous mobile phase A and 50% organic mobile phase B. The block source temperature was maintained at 550 °C and gas values were set as follows: curtain gas 40 psi, GS1 50 psi, GS2 50 psi, and collision-activated dissociation (CAD) gas pressure set as medium. Other parameters were ion spray voltage (−3.5 kV), settling time (15 ms), pause between mass range (5 ms) entrance potential (10 V), collision exit potential (15 V). Acquisition was performed using tandem MS in scheduled selected reaction monitoring mode (Scheduled MRM^TM^ algorithm) by monitoring two transition reactions per compound with an acquisition window of 60 s and a target scan time of 250 ms. Declustering potentials (DP) and collision energies (CE) along with the respective transition reactions and retention time (RT) of each analyte are shown in Table 6. Data acquisition was carried out using Analyst software 1.5.2 and subsequent data processing done using Multiquant software 3.0 (both from Sciex, Foster City, CA, USA).

### 4.6. Identification Criteria

Analytes were considered as positively identified when the following criteria were met simultaneously [33]: (1) a signal visible at the two diagnostic transition reactions monitored for each compound as well as at those of the related internal standard; (2) the retention time of each analyte and each internal standard in the sample extract corresponds to that of the average of the calibration solutions measured in the same sequence with a tolerance of ±0.1 min; (3) the peak area ratio from the different transition reactions recorded for each analyte in the sample extract corresponds to that of the average of the calibration solutions measured in the same sequence with a tolerance of ±30%. Transitions reactions monitored as well as peak area ratio tolerances are given in Table 6.

### 4.7. Quantification

*Alternaria* toxins were quantified by isotopic dilution using seven calibration levels ranging from 0 to 100 ng/mL (0–500 ng/mL for TeA) in water:methanol (80:20) (each level containing internal standard at 10 ng/mL for ALT, AOH, TEN, AME and 50 ng/mL for TeA). An extended calibration curve was applied for TeA in contaminated samples by adding two more calibration levels (1000 and 2000 ng/mL).

A graph “analyte/IS area ratio” on the *y*-axis versus “analyte/IS concentration ratio” on the *x*-axis was plotted for each analyte. A 1/x weighing factor was used to improve precision at the lowest calibration points. Linearity of responses was ensured by checking that the regression coefficient R^2^ was higher than 0.98 and that the deviation of the back-calculated concentration of the calibrants standards from the true concentration was not more than ±20% [33].

The final equation to express each analyte (in μg/kg) was as follows:

(1) $$W=\frac{\left(\frac{A_{a}}{A_{IS}}\right)-I}{S}\times \frac{m_{IS}}{m_{a}}$$

where *A_a_* is the peak area of the analyte in the sample; *A_IS_* is the peak area of the internal standard in the sample; *I* and *S* are the intercept and slope of the regression line, respectively; *m_IS_* is the mass of internal standard added to the test portion in ng (i.e., 50 ng for ALT-IS, AOH-IS, AME-IS, TEN-IS and 250 ng for TeA-IS); *m_a_* is the mass of the test portion in g (i.e., 5, 2.5, or 0.5 g).

### 4.8. Method Validation

Two laboratories were involved in this study and were equipped with the same instrumentation. Laboratory 1 (Switzerland) developed and extensively validated the method whilst Laboratory 2 (Singapore) performed a method verification using the developed protocol without any modifications.

Validation was performed following SANTE/12682/2019 guidelines [33]. Precision data were collected on six different samples representative of each of the six commodity groups (cereal-based products, tomato-based products, tree nuts, vegetable oils, dried fruits, and spices) at one or two fortification levels. Two operators were involved in these experiments, each performing two replicates at the mentioned fortification levels over six different days (leading to a total of 12 separate experiments for each fortification level). Non-fortified samples were also analyzed in duplicate to check for any potential occurrence. For naturally contaminated samples, the native concentration obtained from unspiked samples was tentatively subtracted from the spiked values obtained.

Recovery, repeatability SD(r), and intermediate reproducibility SD(iR) precision data were calculated according to the ISO 5725–2 document [42]. Recovery values at the fortified concentrations were calculated from the median of spiked experiments performed under iR conditions. The overall uncertainty at each fortification level was obtained by combining precision and recovery contributions [43]:

(2) $$U\left(\%\right)=2\times \sqrt{RSD{\left(iR\right)}^{2}+RSD{\left(Rec\right)}^{2}}$$

where *U*(%) is the expanded uncertainty at the 95% confidence interval; *RSD*(*iR*) is the relative standard deviation of intermediate reproducibility and *RSD*(*Rec*) is the relative standard deviation of recovery.

Limits of quantification (LOQs) were set at the lowest validated fortification levels.

The stability of each individual stock standard solution prepared in MeOH (at 100 µg/mL) was checked over 1 year when stored at −20 °C. Each solution was compared to freshly prepared stock standard solutions. The overtime stability of sample extracts was assessed by re-injecting a series of sample extracts, prepared in duplicates, and spiked at two levels, (breakfast cereal, tomato pulp, and chili powder) previously left on the autosampler at 8 °C for 4 days.

Applicability of the method was further demonstrated on a larger range of samples (cocoa, green coffee, herbs, and tea): each sample was fortified at the LOQ and analyzed in duplicate. RSD (%) and recovery (%) were assessed.

Method performance characteristics were verified by Laboratory 2 at one fortification level (corresponding to method LOQs) for four commodity groups (cereal-based products, tomato-based products, vegetable oil, and spice). Three operators were involved, each performing two replicates per fortification level on two occasions (leading to a total of 12 separate experiments for each fortification level).

## Figures and Tables

**Table 1:** Limits of quantification (LOQs) of the five *Alternaria* toxins in various food commodities.

Food Commodity	Analyte	LOQ (µg/kg)
Cereals and cereal-based products, green coffee	ALT, AOH, AME, TEN	0.5
	TeA	2.5
Tree nuts, dried fruits, cocoa, vegetable oil, tomato-based products	ALT, AOH, AME, TEN	2
	TeA	10
Spices, herbs, tea	ALT, AOH, AME, TEN	10
	TeA	50

**Table 2:** Method performance characteristics obtained by Laboratory 1 (Switzerland) and Laboratory 2 (Singapore).

Sample	Lab	Analyte	Fortification Level (µg/kg)	RSD_r_ (%)	RSD_IR_ (%)	Recovery (%)	Measurement Uncertainty (%)
Cereal-based products	Lab 1	ALT	0.5	3.4	5.8	104	12
			25	0.3	4.4	95	11
		AOH	0.5	4.3	12	110	25
			25	1.7	3.9	97	8.4
		AME	0.5	1.2	4.5	104	9.6
			25	0.6	9.1	91	22
		TEN	0.5	5.0	3.7	100	7.4
			25	1.0	1.4	97	4.2
		TeA	2.5	5.7	6.8	109	16
			125	0.4	1.1	105	5.2
	Lab 2	ALT	0.5	5.5	13.0	96	28
		AOH	0.5	3.3	12.7	96	27
		AME	0.5	6.7	14.7	95	31
		TEN	0.5	5.3	6.6	100	14
		TeA	2.5	2.5	5.3	101	11
Tomato-based products	Lab 1	ALT	2	5.1	6.1	102	13
			50	2.8	7.8	94	17
		AOH	2	7.2	9.5	102	20
			50	2.8	2.9	94	8.7
		AME	2	1.9	3.5	101	7.6
			50	0.6	6.3	89	18
		TEN	2	1.2	2.0	99	4.2
			50	0.8	0.8	96	4.0
		TeA	10	5.2	8.4	102	18
			250	0.3	1.4	97	4.5
	Lab 2	ALT	2	4.8	6.4	98	14
		AOH	2	4.5	6.5	99	14
		AME	2	1.2	3.1	109	10
		TEN	2	2.1	1.9	110	10
		TeA	10	7.7	12	106	26
Spices	Lab 1	ALT	10	6.6	6.0	100	12
			250	2.3	4.4	90	15
		AOH	10	7.9	12	101	25
			250	4.6	7.8	93	18
		AME	10	3.2	15	106	32
			250	0.9	3.5	94	10
		TEN	10	2.4	2.4	110	10
			250	1.1	1.1	97	3.5
		TeA	1250	26	29	91	58
	Lab 2	ALT	10	7.2	16	98	34
		AOH	10	3.9	5.6	98	12
		AME	10	1.4	4.4	113	15
		TEN	10	3.1	4.0	115	16
		TeA	50	19	19	100	40
Sunflower oil	Lab 1	ALT	2	4.7	3.9	102	8.3
		AOH	2	3.2	3.3	102	7.0
		AME	2	1.5	2.8	102	5.9
		TEN	2	1.4	2.1	98	4.4
		TeA	10	1.7	2.4	103	5.8
	Lab 2	ALT	2	2.4	4.9	98	10
		AOH	2	3.3	4.7	95	11
		AME	2	1.1	9.9	92	21
		TEN	2	2.4	2.5	100	5.1
		TeA	10	1.0	1.4	97	4.2
Dried fruits	Lab 1	ALT	2	4.1	11	92	23
		AOH	2	12	11	106	23
		AME	2	2.2	3.5	94	9.9
		TEN	2	1.7	3.0	103	6.4
		TeA	10	2.9	3.2	95	8.7
Tree nuts	Lab 1	ALT	2	6.3	8.4	106	18
		AOH	2	7.8	7.3	95	16
		AME	2	2.0	7.4	101	16
		TEN	2	1.6	1.9	103	4.7
		TeA	10	5.7	5.3	112	15

**Table 3:** Occurrence of *Alternaria* toxins in 138 food items. Ranges of concentrations are given in µg/kg (nb of positives).

Foods (Nb of Samples)	ALT	AOH	AME	TEN	TeA
Cereals (31)	<0.5 (0)	<0.5–11.8 (8)	<0.5–3.4 (6)	<0.5–31.2 (9)	<2.5–766 (25)
Cereal-based products (15)	<0.5 (0)	<0.5–7.4 (4)	<0.5–2.7 (3)	<0.5–6.9 (10)	<2.5–628 (11)
Cocoa (5)	<2 (0)	<2 (0)	<2 (0)	<2 (0)	<10 (0)
Fruits, dried and juice (9)	<2–17.2 (1)	<2–80.2 (3)	<2–47.8 (2)	<2–13.4 (1)	<10–685 (7)
Herbs (11)	<10 (0)	<10–111 (6)	<10–25.6 (4)	<10–113 (5)	<50–748 (10)
Nuts (13)	<2 (0)	<2–6.4 (4)	<2–3.5 (2)	<2 (0)	<10–62.0 (4)
Sunflower oil (4)	<2 (0)	<2 (0)	<2–2.4 (1)	<2–3.9 (1)	<10 (0)
Spices (21)	<10 (0)	<10–153 (13)	<10–73.6 (10)	<10–73.4 (13)	<50–20,478 (19)
Tea (15)	<10 (0)	<10 (0)	<10 (0)	<10 (0)	<50 (0)
Vegetables incl. tomato (14)	<2 (0)	<2–65.3 (7)	<2–7.9 (1)	<2–3.1 (2)	<10–1096 (11)

**Table 4:** Occurrence of *Alternaria* toxins in green coffee (*n* = 78) from 21 producing countries. Range of concentrations is given in µg/kg (nb of positive).

Producing Countries (Nb of Samples)	ALT	AOH	AME	TEN	TeA
Brazil (5)	<0.5 (0)	<0.5 (0)	<0.5 (0)	<0.5 (0)	<2.5–10.0 (1)
Cameroon (2)	<0.5 (0)	<0.5 (0)	<0.5 (0)	<0.5 (0)	<2.5 (0)
China (2)	<0.5 (0)	<0.5 (0)	<0.5 (0)	<0.5 (0)	<2.5 (0)
Columbia (4)	<0.5 (0)	<0.5 (0)	<0.5 (0)	<0.5 (0)	<2.5 (0)
Costa Rica (2)	<0.5 (0)	<0.5 (0)	<0.5 (0)	<0.5 (0)	<2.5 (0)
Ethiopia (4)	<0.5 (0)	<0.5 (0)	<0.5 (0)	<0.5 (0)	<2.5 (0)
Guatemala (2)	<0.5 (0)	<0.5 (0)	<0.5 (0)	<0.5 (0)	<2.5 (0)
Honduras (4)	<0.5 (0)	<0.5 (0)	<0.5 (0)	<0.5 (0)	<2.5 (0)
India (2)	<0.5 (0)	<0.5 (0)	<0.5 (0)	<0.5 (0)	<2.5 (0)
Indonesia (4)	<0.5 (0)	<0.5 (0)	<0.5 (0)	<0.5 (0)	<2.5 (0)
Ivory Coast (6)	<0.5 (0)	<0.5–1.2 (1)	<0.5 (0)	<0.5 (0)	<2.5 (0)
Kenya (2)	<0.5 (0)	<0.5 (0)	<0.5 (0)	<0.5 (0)	<2.5 (0)
Mexico (3)	<0.5 (0)	<0.5 (0)	<0.5 (0)	<0.5 (0)	<2.5 (0)
Nicaragua (2)	<0.5 (0)	<0.5 (0)	<0.5 (0)	<0.5 (0)	<2.5 (0)
Papua New Guinea (4)	<0.5 (0)	<0.5 (0)	<0.5 (0)	<0.5 (0)	<2.5 (0)
Peru (4)	<0.5 (0)	<0.5 (0)	<0.5 (0)	<0.5 (0)	<2.5–13.2 (1)
Philippines (2)	<0.5 (0)	<0.5 (0)	<0.5 (0)	<0.5 (0)	<2.5 (0)
Rwanda (2)	<0.5 (0)	<0.5 (0)	<0.5 (0)	<0.5 (0)	<2.5 (0)
Thailand (2)	<0.5 (0)	<0.5 (0)	<0.5 (0)	<0.5 (0)	<2.5 (0)
Uganda (4)	<0.5 (0)	<0.5 (0)	<0.5 (0)	<0.5 (0)	<2.5 (0)
Vietnam (16)	<0.5 (0)	<0.5–1.7 (3)	<0.5–1.3 (1)	<0.5–0.5 (1)	<2.5 (0)

**Table 5:** Summary of occurrence of *Alternaria* toxins in 78 green coffee samples, and exposure from highest levels recorded.

Toxin	LOQ (µg/kg)	Number of Samples			Max Level (µg/kg)	Max Exposure ^2^ (ng/kg BW/d)	TTC (ng/kg BW/d)	% TTC ^2^
		ND ^1^	<LOQ	>LOQ				
ALT	0.5	78 (100%)	0	0	-	-	1500	-
AOH	0.5	73 (94%)	1 (1%)	4 (5%)	2.75	1.3	2.5	52
AME	0.5	71 (91%)	6 (8%)	1 (1%)	1.29	0.6	2.5	24
TEN	0.5	77 (99%)	0	1 (1%)	0.52	0.2	1500	0.01
TeA	2.5	75 (96%)	1 (1%)	2 (3%)	13.2	6.2	1500	0.4
AOH + AME	-	-	-	-	2.98	1.4	2.5	56

^1^ Not detected; ^2^ exposure and %TTC resulting from the highest concentration level of each toxin analyzed.

**Table 6:** MS/MS parameters for *Alternaria* toxins in negative electrospray ionization (ESI) mode (collision energy for each transition reaction is given in brackets).

Analyte	RT (min)	DP (V)	Quantification (*m/z*)	Confirmation (*m/z*)	Peak Area Ratio
ALT	3.45	−60	291.0 > 214.1 (−30)	291.0 > 186.1 (−35)	0.66
ALT-IS	3.45	−45	297.0 > 217.2 (−30)	297.0 > 189.0 (−38)	0.77
AOH	3.80	−60	257.0 > 215.1 (−35)	257.0 > 212.0 (−40)	0.60
AOH-IS	3.80	−65	260.0 > 218.0 (−35)	260.0 > 215.1 (−40)	0.94
AME	4.95	−60	271.0 > 256.0 (−30)	271.0 > 228.0 (−40)	0.27
AME-IS	4.95	−60	274.0 > 259.0 (−30)	274.0 > 231.1 (−40)	0.30
TEN	4.25	−40	413.2 > 141.0 (−25)	413.2 > 271.0 (−22)	0.69
TEN-IS	4.25	−45	416.2 > 141.0 (−27)	416.2 > 274.1 (−23)	0.67
TeA	2.05	−40	196.1 > 139.1 (−25)	196.1 > 112.1 (−30)	0.74
TeA-IS	2.05	−45	198.1 > 141.0 (−25)	198.1 > 114.0 (−32)	0.79

## Supplementary Materials

The following are available online at https://www.mdpi.com/2072-6651/12/9/595/s1, Table S1: Individual levels of *Alternaria* toxins in 216 samples.
