# Supplementary material for: Levels of Alternaria Toxins in Selected Food Commodities Including Green Coffee
Source: Toxins (Basel). 2020 Sep 15;12(9):595. doi: 10.3390/toxins12090595 (PMC7551213; doi:10.3390/toxins12090595)
Supplement: Supplementary file 1 [file toxins-12-00595-s001.pdf]

Supplementary Materials: Levels of *Alternaria* Toxins in Selected Food Commodities Including Green Coffee

Claudia Mujahid, Marie-Claude Savoy, Quentin Baslé, Pei Mun Woo, Edith Chin Yean Ee, Pascal Mottier and Thomas Bessaire

Table S1. Individual levels of five *Alternaria* toxins in 216 samples (values in μg/kg).

Category	Sample Name	ALT	AOH	AME	TEN	TeA
Cereal-based foods	Biscuits_1	< 0.5	< 0.5	< 0.5	1.5	5.2
Cereal-based foods	Biscuits_2	< 0.5	< 0.5	< 0.5	1.2	< 2.5
Cereal-based foods	Biscuits_3	< 0.5	< 0.5	< 0.5	0.9	6.6
Cereal-based foods	Biscuits_4	< 0.5	< 0.5	< 0.5	< 0.5	< 2.5
Cereal-based foods	Biscuits_5	< 0.5	< 0.5	< 0.5	6.9	29.6
Cereal-based foods	Biscuits_6	< 0.5	< 0.5	< 0.5	< 0.5	< 2.5
Cereal-based foods	Biscuits_7	< 0.5	2.0	0.9	< 0.5	< 2.5
Cereal-based foods	Breakfast cereal_1	< 0.5	0.8	< 0.5	0.8	20.7
Cereal-based foods	Breakfast cereal_2	< 0.5	< 0.5	< 0.5	< 0.5	35.0
Cereal-based foods	Breakfast cereal_3	< 0.5	< 0.5	< 0.5	1.5	6.9
Cereal-based foods	Breakfast cereal_4	< 0.5	< 0.5	< 0.5	< 0.5	13.1
Cereal-based foods	Breakfast cereal_5	< 0.5	< 0.5	< 0.5	0.9	9.4
Cereal-based foods	Breakfast cereal_6	< 0.5	< 0.5	< 0.5	0.8	30.3
Cereal-based foods	Breakfast cereal_7	< 0.5	7.4	2.7	6.3	628
Cereal-based foods	Breakfast cereal_8	< 0.5	3.2	1.1	2.7	324
Cereals	Barley_1	< 0.5	< 0.5	< 0.5	< 0.5	< 2.5
Cereals	Barley_2	< 0.5	< 0.5	< 0.5	4.6	52.6
Cereals	Barley_3	< 0.5	2.2	< 0.5	6.2	67.2
Cereals	Corn_1	< 0.5	< 0.5	< 0.5	< 0.5	4.0
Cereals	Corn_2	< 0.5	< 0.5	< 0.5	< 0.5	3.5
Cereals	Millet_1	< 0.5	1.0	< 0.5	< 0.5	186
Cereals	Millet_2	< 0.5	< 0.5	< 0.5	< 0.5	< 2.5
Cereals	Oat	< 0.5	< 0.5	< 0.5	0.5	< 2.5
Cereals	Rapeseed_1	< 0.5	11.7	3.4	31.2	766
Cereals	Rapeseed_2	< 0.5	11.8	3.2	29.0	636
Cereals	Rice_1	< 0.5	< 0.5	< 0.5	< 0.5	27.8
Cereals	Rice_2	< 0.5	< 0.5	< 0.5	< 0.5	17.4
Cereals	Rice_3	< 0.5	< 0.5	< 0.5	< 0.5	11.6
Cereals	Rice_4	< 0.5	< 0.5	< 0.5	< 0.5	38.2
Cereals	Rice_5	< 0.5	< 0.5	< 0.5	< 0.5	8.0
Cereals	Rice_6	< 0.5	1.1	1.5	< 0.5	758
Cereals	Rice_7	< 0.5	< 0.5	< 0.5	< 0.5	17.0
Cereals	Rice_8	< 0.5	< 0.5	< 0.5	< 0.5	78.2
Cereals	Rice_9	< 0.5	< 0.5	< 0.5	< 0.5	10.1
Cereals	Rice_10	< 0.5	< 0.5	< 0.5	< 0.5	< 2.5
Cereals	Rice_11	< 0.5	< 0.5	< 0.5	< 0.5	72.6
Cereals	Rice_12	< 0.5	< 0.5	< 0.5	< 0.5	< 2.5
Cereals	Rye	< 0.5	< 0.5	< 0.5	< 0.5	< 2.5
Cereals	Sorghum	< 0.5	< 0.5	< 0.5	< 0.5	28.9

Category	Sample Name	ALT	AOH	AME	TEN	TeA
Cereals	Soya_1	< 0.5	1.8	1.2	20.1	207
Cereals	Soya_2	< 0.5	3.0	1.8	30.8	365
Cereals	Spelt	< 0.5	< 0.5	< 0.5	< 0.5	8.0
Cereals	Triticale	< 0.5	< 0.5	< 0.5	< 0.5	< 2.5
Cereals	Wheat_1	< 0.5	< 0.5	< 0.5	< 0.5	< 2.5
Cereals	Wheat_2	< 0.5	9.7	1.8	8.0	36.4
Cereals	Wheat_3	< 0.5	< 0.5	< 0.5	3.9	69.4
Cocoa	Cocoa Nibs_1	< 2	< 2	< 2	< 2	< 10
Cocoa	Cocoa Nibs_2	< 2	< 2	< 2	< 2	< 10
Cocoa	Cocoa Powder_1	< 2	< 2	< 2	< 2	< 10
Cocoa	Cocoa Powder_2	< 2	< 2	< 2	< 2	< 10
Cocoa	Cocoa Powder_3	< 2	< 2	< 2	< 2	< 10
Fruits	Apricots (dried)_1	< 2	< 2	< 2	< 2	61.0
Fruits	Apricots (dried)_2	< 2	< 2	< 2	< 2	59.8
Fruits	Blueberries (dried)_1	< 2	5.4	< 2	< 2	129
Fruits	Blueberries (dried)_2	< 2	19.6	5.7	< 2	158
Fruits	Pomegrenate Juice (conc)	17.2	80.2	47.8	13.4	685
Fruits	Raisins (dried)_1	< 2	< 2	< 2	< 2	47.4
Fruits	Raisins (dried)_2	< 2	< 2	< 2	< 2	33.2
Fruits	Raisins (dried)_3	< 2	< 2	< 2	< 2	< 10
Fruits	Raisins (dried)_4	< 2	< 2	< 2	< 2	< 10
Herbs	Marjoram_1	< 10	< 10	< 10	25.1	265
Herbs	Marjoram_2	< 10	13.0	< 10	32.6	353
Herbs	Marjoram_3	< 10	< 10	< 10	< 10	< 50
Herbs	Marjoram_4	< 10	< 10	< 10	< 10	69.9
Herbs	Marjoram_5	< 10	23.8	13.0	113	456
Herbs	Marjoram_6	< 10	< 10	< 10	< 10	81.2
Herbs	Oregano_1	< 10	96.3	25.6	38.3	343
Herbs	Oregano_2	< 10	70.2	17.4	64.4	115
Herbs	Thyme_1	< 10	111	19.5	< 10	137
Herbs	Thyme_2	< 10	46.3	< 10	< 10	748
Herbs	Thyme_3	< 10	< 10	< 10	< 10	287
Nuts	Almonds_1	< 2	< 2	< 2	< 2	< 10
Nuts	Almonds_2	< 2	< 2	< 2	< 2	< 10
Nuts	Almonds_3	< 2	< 2	< 2	< 2	< 10
Nuts	Almonds_4	< 2	< 2	< 2	< 2	< 10
Nuts	Almonds_5	< 2	< 2	< 2	< 2	< 10
Nuts	Hazelnuts_1	< 2	< 2	< 2	< 2	40.0
Nuts	Hazelnuts_2	< 2	3.8	< 2	< 2	< 10
Nuts	Hazelnuts_3	< 2	3.1	3.5	< 2	62.0
Nuts	Peanuts_1	< 2	2.9	2.8	< 2	36.0
Nuts	Peanuts_2	< 2	< 2	< 2	< 2	< 10
Nuts	Peanuts_3	< 2	< 2	< 2	< 2	< 10
Nuts	Pistachio_1	< 2	< 2	< 2	< 2	44.0
Nuts	Pistachio_2	< 2	6.4	< 2	< 2	< 10
Spices	Caraway_1	< 10	16.7	< 10	42.0	95
Spices	Caraway_2	< 10	25.2	< 10	24.8	98

Category	Sample Name	ALT	AOH	AME	TEN	TeA
Spices	Caraway_3	< 10	< 10	< 10	< 10	83
Spices	Cayenne pepper_1	< 10	< 10	< 10	< 10	5615
Spices	Chili_1	< 10	153	66.0	< 10	9991
Spices	Chili_2	< 10	59.3	17.0	21.8	20478
Spices	Chili_3	< 10	20.8	15.0	32.9	10676
Spices	Chili_4	< 10	26.3	10.1	26.8	18555
Spices	Chili_5	< 10	< 10	< 10	< 10	4510
Spices	Coriander_1	< 10	< 10	< 10	< 10	249
Spices	Coriander_2	< 10	< 10	< 10	< 10	228
Spices	Garlic_1	< 10	< 10	< 10	< 10	< 50
Spices	Garlic_2	< 10	< 10	< 10	< 10	< 50
Spices	Paprika_1	< 10	< 10	< 10	19.5	10186
Spices	Paprika_2	< 10	66.4	40.8	73.4	14509
Spices	Paprika_3	< 10	74.3	48.7	70.8	15350
Spices	Paprika_4	< 10	23.5	11.5	30.9	13593
Spices	Paprika_5	< 10	21.1	< 10	32.8	18856
Spices	Paprika_6	< 10	121	73.6	36.8	10163
Spices	Paprika_7	< 10	62.5	16.1	27.6	18242
Spices	Paprika_8	< 10	23.9	16.7	37.5	7356
Oil	Sunflower Oil_1	< 2	< 2	< 2	3.9	< 10
Oil	Sunflower Oil_2	< 2	< 2	2.4	< 2	< 10
Oil	Sunflower Oil_3	< 2	< 2	< 2	< 2	< 10
Oil	Sunflower Oil_4	< 2	< 2	< 2	< 2	< 10
Tea	Black Tea_1	< 10	< 10	< 10	< 10	< 50
Tea	Black Tea_2	< 10	< 10	< 10	< 10	< 50
Tea	Black Tea_3	< 10	< 10	< 10	< 10	< 50
Tea	Black Tea_4	< 10	< 10	< 10	< 10	< 50
Tea	Black Tea_5	< 10	< 10	< 10	< 10	< 50
Tea	Blue Tea	< 10	< 10	< 10	< 10	< 50
Tea	Green Tea_1	< 10	< 10	< 10	< 10	< 50
Tea	Green Tea_2	< 10	< 10	< 10	< 10	< 50
Tea	Green Tea_3	< 10	< 10	< 10	< 10	< 50
Tea	Green Tea_4	< 10	< 10	< 10	< 10	< 50
Tea	Green Tea_5	< 10	< 10	< 10	< 10	< 50
Tea	Green Tea_6	< 10	< 10	< 10	< 10	< 50
Tea	Green Tea_7	< 10	< 10	< 10	< 10	< 50
Tea	Green Tea_8	< 10	< 10	< 10	< 10	< 50
Tea	White Tea	< 10	< 10	< 10	< 10	< 50
Tomato products	Ketchup	< 2	< 2	< 2	< 2	30.5
Tomato products	Concentrate_1	< 2	2.9	< 2	< 2	162
Tomato products	Concentrate_2	< 2	65.3	7.9	< 2	1096
Tomato products	Dried_1	< 2	< 2	< 2	< 2	270
Tomato products	Dried_2	< 2	< 2	< 2	< 2	153
Tomato products	Dried_3	< 2	13.7	< 2	< 2	641
Tomato products	Puree_1	< 2	< 2	< 2	< 2	30.0
Tomato products	Puree_2	< 2	3.2	< 2	< 2	60.3
Tomato products	Puree_3	< 2	7.6	< 2	< 2	46.7
Other vegetables	Carrot_1	< 2	5.6	< 2	2.4	12.5

Category	Sample Name	ALT	AOH	AME	TEN	TeA
Other vegetables	Carrot_2	< 2	8.1	< 2	3.1	23.5
Other vegetables	Pea	< 2	< 2	< 2	< 2	< 10
Other vegetables	Pea Proteins_1	< 2	< 2	< 2	< 2	< 10
Other vegetables	Pea Proteins_2	< 2	< 2	< 2	< 2	< 10
Green coffee	Brazil_1	< 0.5	< 0.5	< 0.5	< 0.5	< 2.5
Green coffee	Brazil_2	< 0.5	< 0.5	< 0.5	< 0.5	9.96
Green coffee	Brazil_3	< 0.5	< 0.5	< 0.5	< 0.5	< 2.5
Green coffee	Brazil_4	< 0.5	< 0.5	< 0.5	< 0.5	< 2.5
Green coffee	Brazil_5	< 0.5	< 0.5	< 0.5	< 0.5	< 2.5
Green coffee	Cameroon_1	< 0.5	< 0.5	< 0.5	< 0.5	< 2.5
Green coffee	Cameroon_2	< 0.5	< 0.5	< 0.5	< 0.5	< 2.5
Green coffee	China_1	< 0.5	< 0.5	< 0.5	< 0.5	< 2.5
Green coffee	China_2	< 0.5	< 0.5	< 0.5	< 0.5	< 2.5
Green coffee	Colombia_1	< 0.5	< 0.5	< 0.5	< 0.5	< 2.5
Green coffee	Colombia_2	< 0.5	< 0.5	< 0.5	< 0.5	< 2.5
Green coffee	Colombia_3	< 0.5	< 0.5	< 0.5	< 0.5	< 2.5
Green coffee	Colombia_4	< 0.5	< 0.5	< 0.5	< 0.5	< 2.5
Green coffee	Costa Rica_1	< 0.5	< 0.5	< 0.5	< 0.5	< 2.5
Green coffee	Costa Rica_2	< 0.5	< 0.5	< 0.5	< 0.5	< 2.5
Green coffee	Ethiopia_1	< 0.5	< 0.5	< 0.5	< 0.5	< 2.5
Green coffee	Ethiopia_2	< 0.5	< 0.5	< 0.5	< 0.5	< 2.5
Green coffee	Ethiopia_3	< 0.5	< 0.5	< 0.5	< 0.5	< 2.5
Green coffee	Ethiopia_4	< 0.5	< 0.5	< 0.5	< 0.5	< 2.5
Green coffee	Guatemala_1	< 0.5	< 0.5	< 0.5	< 0.5	< 2.5
Green coffee	Guatemala_2	< 0.5	< 0.5	< 0.5	< 0.5	< 2.5
Green coffee	Honduras_1	< 0.5	< 0.5	< 0.5	< 0.5	< 2.5
Green coffee	Honduras_2	< 0.5	< 0.5	< 0.5	< 0.5	< 2.5
Green coffee	Honduras_3	< 0.5	< 0.5	< 0.5	< 0.5	< 2.5
Green coffee	Honduras_4	< 0.5	< 0.5	< 0.5	< 0.5	< 2.5
Green coffee	India_1	< 0.5	< 0.5	< 0.5	< 0.5	< 2.5
Green coffee	India_2	< 0.5	< 0.5	< 0.5	< 0.5	< 2.5
Green coffee	Indonesia_1	< 0.5	< 0.5	< 0.5	< 0.5	< 2.5
Green coffee	Indonesia_2	< 0.5	< 0.5	< 0.5	< 0.5	< 2.5
Green coffee	Indonesia_3	< 0.5	< 0.5	< 0.5	< 0.5	< 2.5
Green coffee	Indonesia_4	< 0.5	< 0.5	< 0.5	< 0.5	< 2.5
Green coffee	Ivory Coast_1	< 0.5	1.24	< 0.5	< 0.5	< 2.5
Green coffee	Ivory Coast_2	< 0.5	< 0.5	< 0.5	< 0.5	< 2.5
Green coffee	Ivory Coast_3	< 0.5	< 0.5	< 0.5	< 0.5	< 2.5
Green coffee	Ivory Coast_4	< 0.5	< 0.5	< 0.5	< 0.5	< 2.5
Green coffee	Ivory Coast_5	< 0.5	< 0.5	< 0.5	< 0.5	< 2.5
Green coffee	Ivory Coast_6	< 0.5	< 0.5	< 0.5	< 0.5	< 2.5
Green coffee	Kenya_1	< 0.5	< 0.5	< 0.5	< 0.5	< 2.5
Green coffee	Kenya_2	< 0.5	< 0.5	< 0.5	< 0.5	< 2.5
Green coffee	Mexico_1	< 0.5	< 0.5	< 0.5	< 0.5	< 2.5
Green coffee	Mexico_2	< 0.5	< 0.5	< 0.5	< 0.5	< 2.5
Green coffee	Mexico_3	< 0.5	< 0.5	< 0.5	< 0.5	< 2.5
Green coffee	Nicaragua_1	< 0.5	< 0.5	< 0.5	< 0.5	< 2.5
Green coffee	Nicaragua_2	< 0.5	< 0.5	< 0.5	< 0.5	< 2.5

**Category**	**Sample Name**	**ALT**	**AOH**	**AME**	**TEN**	**TeA**
Green coffee	Papua New Guinea_1	< 0.5	< 0.5	< 0.5	< 0.5	< 2.5
Green coffee	Papua New Guinea_2	< 0.5	< 0.5	< 0.5	< 0.5	< 2.5
Green coffee	Papua New Guinea_3	< 0.5	< 0.5	< 0.5	< 0.5	< 2.5
Green coffee	Papua New Guinea_4	< 0.5	< 0.5	< 0.5	< 0.5	< 2.5
Green coffee	Peru_1	< 0.5	< 0.5	< 0.5	< 0.5	< 2.5
Green coffee	Peru_2	< 0.5	< 0.5	< 0.5	< 0.5	< 2.5
Green coffee	Peru_3	< 0.5	< 0.5	< 0.5	< 0.5	13.2
Green coffee	Peru_4	< 0.5	< 0.5	< 0.5	< 0.5	< 2.5
Green coffee	Philippines_1	< 0.5	< 0.5	< 0.5	< 0.5	< 2.5
Green coffee	Philippines_2	< 0.5	< 0.5	< 0.5	< 0.5	< 2.5
Green coffee	Rwanda_1	< 0.5	< 0.5	< 0.5	< 0.5	< 2.5
Green coffee	Rwanda_2	< 0.5	< 0.5	< 0.5	< 0.5	< 2.5
Green coffee	Thailand_1	< 0.5	< 0.5	< 0.5	< 0.5	< 2.5
Green coffee	Thailand_2	< 0.5	< 0.5	< 0.5	< 0.5	< 2.5
Green coffee	Uganda_1	< 0.5	< 0.5	< 0.5	< 0.5	< 2.5
Green coffee	Uganda_2	< 0.5	< 0.5	< 0.5	< 0.5	< 2.5
Green coffee	Uganda_3	< 0.5	< 0.5	< 0.5	< 0.5	< 2.5
Green coffee	Uganda_4	< 0.5	< 0.5	< 0.5	< 0.5	< 2.5
Green coffee	Vietnam_1	< 0.5	2.75	< 0.5	< 0.5	< 2.5
Green coffee	Vietnam_2	< 0.5	0.72	< 0.5	< 0.5	< 2.5
Green coffee	Vietnam_3	< 0.5	< 0.5	< 0.5	< 0.5	< 2.5
Green coffee	Vietnam_4	< 0.5	< 0.5	< 0.5	< 0.5	< 2.5
Green coffee	Vietnam_5	< 0.5	< 0.5	< 0.5	< 0.5	< 2.5
Green coffee	Vietnam_6	< 0.5	< 0.5	< 0.5	< 0.5	< 2.5
Green coffee	Vietnam_7	< 0.5	< 0.5	< 0.5	< 0.5	< 2.5
Green coffee	Vietnam_8	< 0.5	< 0.5	< 0.5	< 0.5	< 2.5
Green coffee	Vietnam_9	< 0.5	< 0.5	< 0.5	< 0.5	< 2.5
Green coffee	Vietnam_10	< 0.5	< 0.5	< 0.5	< 0.5	< 2.5
Green coffee	Vietnam_11	< 0.5	1.69	1.29	< 0.5	< 2.5
Green coffee	Vietnam_12	< 0.5	< 0.5	< 0.5	< 0.5	< 2.5
Green coffee	Vietnam_13	< 0.5	< 0.5	< 0.5	< 0.5	< 2.5
Green coffee	Vietnam_14	< 0.5	< 0.5	< 0.5	0.52	< 2.5
Green coffee	Vietnam_15	< 0.5	< 0.5	< 0.5	< 0.5	< 2.5
Green coffee	Vietnam_16	< 0.5	< 0.5	< 0.5	< 0.5	< 2.5
